# Structural and antimicrobial properties of synthesized gold nanoparticles using biological and chemical approaches

**DOI:** 10.3389/fchem.2024.1482102

**Published:** 2024-11-13

**Authors:** Hamidreza Kalantari, Raymond J. Turner

**Affiliations:** ^1^ Department of Biological Sciences, Microbial Biochemistry Laboratory, University of Calgary, Calgary, NW, Canada; ^2^ Department of Microbiology, Islamic Azad University, Tehran, Iran

**Keywords:** gold nanoparticles (AuNPs), green synthesis, ginger AuNPs, curcumin AuNPs, chemical AuNPs (sodium citrate), antimicrobial activity, nanoparticle characterization

## Abstract

This study explores the synthesis and characterization of gold nanoparticles (AuNPs) using green and chemical methods, employing ginger extract and curcumin as reducing agents, in comparison to sodium citrate reduction. The biosynthesized AuNPs synthesized with ginger extract exhibited an average hydrodynamic diameter of 15 and 10 nm for curcumin-conjugated AuNPs, while chemically synthesized AuNPs with sodium citrate displayed an average size of 10 nm. Assessments via Zeta potential measurements revealed negative surface charges across all samples, with the curcumin-conjugated AuNPs showing −36.3 mV, ginger extract-synthesized AuNPs showing −31.7 mV, and chemically produced gold nanoparticles having a surface charge of −40.4 mV. Transmission Electron Microscopy (TEM) confirmed spherical morphologies for the synthesized nanoparticles,and it revealed the presence of biomolecules embedded within the nanoparticles synthesized using biological materials, whereas chemically synthesized AuNPs lacked such features. The FTIR spectra of the biosynthesized AuNPs highlighted the presence of phenolic and aromatic compounds from the ginger extract and curcumin, indicating their role in coating the nanoparticles. Gas chromatography-mass spectrometry (GC-MS) analysis identified gingerol as a key component in the ginger extract, contributing to nanoparticle capping. The antimicrobial efficacy of the AuNPs was evaluated against *P. aeruginosa*, *E. coli*, and *S. aureus*, revealing superior activity for curcumin-AuNPs, with ginger-AuNPs also outperforming chemically synthesized counterparts. These findings confirm the advantages of biological approaches, using a plant extract like ginger and pure curcumin suspension, for better size distribution when used as reducing agents, along with improved antimicrobial efficacy compared to chemically produced gold nanoparticles synthesized with sodium citrate. This study also highlight the potential of green-synthesized AuNPs in biomedical applications, due to their enhanced stability from higher surface charge and the repeatability of biological methods.

## 1 Introduction

Nanoparticles, defined as particles with dimensions between 1 and 100 nm, are of significant interest due to their unique size-dependent properties, which enable diverse applications in fields such as medicine, electronics, and environmental science (Bhattacharyya et al., 2011). Moreover, biogenic gold nanoparticles, produced through environmentally friendly processes using biological organisms, are increasingly studied for their potential applications in drug delivery, diagnostics, and environmental remediation (Singh et al., 2016). Gold nanoparticle research has become a significant field within colloids and surfaces, driven by their unique optical characteristics, size-dependent electrochemical behavior, and exceptional chemical stability (Sardar et al., 2009). Additionally, the utilization of gold nanoparticles (AuNPs) dates back to ancient Roman times, highlighting its enduring significance and diverse applications (Louis and Pluchery, 2017). Gold nanoparticles were used in the Roman era primarily in the creation of the Lycurgus Cup, a remarkable example of ancient nanotechnology (Freestone et al., 2007). Over the past 2 decades, there has been extensive research and evaluation of nanoparticles (NPs) as potential antimicrobial agents (Ankamwar et al., 2005). Initially, the primary focus was on synthesizing nanoparticles (NPs) using various chemical or physical methods. However, the high production costs and the generation of toxic by-products created a demand for innovative synthesis methods for nanoparticles (Ankamwar et al., 2005; Jiménez-González et al., 2004). The increasing need for metal nanoparticles should be met with eco-friendly, cost-effective, and innovative synthesis methods to reduce or eliminate the use of harmful chemicals and minimize hazardous waste accumulation. For instance, dispersants, surfactants, or chelating agents are commonly used in chemically synthesized nanomaterials to inhibit particle agglomeration. When employed in large-scale production, these reagents can become environmental pollutants. Furthermore, the nanomaterials produced through these conventional methods may be associated with instability, bio-incompatibility, and toxicity, hindering their application in both environmental contexts and human health (Ijaz et al., 2020). This reality has created a demand for innovative synthesis methods that are eco-friendly and cost-effective. Safer production alternatives involve using gentle solvents, environmentally friendly reducing or stabilizing agents, mild experimental conditions, or employing biological systems like plant extracts, bacteria, fungi, or their lysates and extracted biomolecules. These sustainable methods are often referred to as green approaches in nanoparticle synthesis (Boroumand Moghaddam et al., 2015; Mittal et al., 2013). Among the diverse biological sources explored for nanomaterial synthesis, plants have emerged as the leader in this regard. Plant extracts contain a rich array of phytochemicals such as alkaloids, terpenes, saponins, phenols, alcohols, and proteins, which serve as both reducing and capping agents in nanoparticle formation (Barabadi et al., 2020).

Some plants, such as ginger and curcumin, exhibit significant antimicrobial activity, which has been demonstrated in various studies (Pormohammad et al., 2022; Teles et al., 2019). Ginger (*Zingiber officinale*) from the Zingiberaceae family is a perennial herb characterized by its robust, tuberous rhizomes. Research conducted by Malu and colleagues reveals that components within ginger extract solutions, using n-hexane or ethyl acetate using a Soxhlet extractor, exhibit significant antibacterial effects of both bacteriostatic and bactericidal activity, highlighting the multifaceted antimicrobial potential of ginger extracts (Malu et al., 2009).

Curcumin, extracted from the rhizome of turmeric, stands as one of the premier cash crops widely cultivated across Asia, and its medicinal use dating back to ancient times (Ciuca and Racovita, 2023) This flavonoid compound, scientifically named 1,7-bis-(4-hydroxy-3-methoxyphenyl)-1,6-heptadiene-2,5-dione, is renowned for its diverse range of therapeutic properties. It is notably recognized for its antioxidant, anti-inflammatory, antimicrobial, anti-carcinogenic, and immunomodulatory activities. These attributes make curcumin a valuable component in traditional medicine and a subject of research towards its potential in treating various ailments (Anand et al., 2007; Teiten et al., 2010).

There are numerous methods for producing gold nanoparticles, including various chemical approaches. One widely used technique involves the chemical reduction of gold ions to form nanoparticles. In this process, sodium citrate acts both as a reducing agent and a coating cap, facilitating the conversion of gold ions (Au⁺³) into elemental gold nanoparticles. The sodium citrate reduces the gold ions to their atom metal state while simultaneously preventing the agglomeration of the nanoparticles, thereby controlling their size and distribution (De Souza et al., 2019; Al-Johani et al., 2017).

In this study, we explore the synthesis of gold nanoparticles through two distinct methodologies: biological and chemical. We employ extracts of ginger and curcumin for the biological synthesis, while sodium citrate is utilized as a reducing agent in the chemical synthesis. Our goal is to compare the properties of gold nanoparticles produced by these methods and assessed their antimicrobial efficacy against *Pseudomonas aeruginosa*, *Escherichia coli*, and *Staphylococcus aureus*. This comparative analysis aims to elucidate how different synthesis techniques influence the characteristics and performance of the nanoparticles, and to determine whether biologically synthesized nanoparticles exhibit superior antimicrobial activity compared to their chemically synthesized counterparts.

## 2 Materials

Fresh Zingiber officinale roots (grown in the Fraser Valley farming area, Canada), hydrogen tetrachloroaurate (III) trihydrate (HAuCl_4_·3H_2_O) (Alfa Aesar), curcumin (95% purity) (Alfa Aesar), sodium hydroxide (NaOH) (Sigma-Aldrich), sodium citrate (38.8 mM) (Sigma-Aldrich), deionized water (produced using a Milli-Q system).

## 3 Methods

### 3.1 Synthesizing gold nanoparticles

#### 3.1.1 Synthesizing gold nanoparticles with the extract of ginger root

##### 3.1.1.1 Preparation of the extract of ginger

**FIGURE 1:**
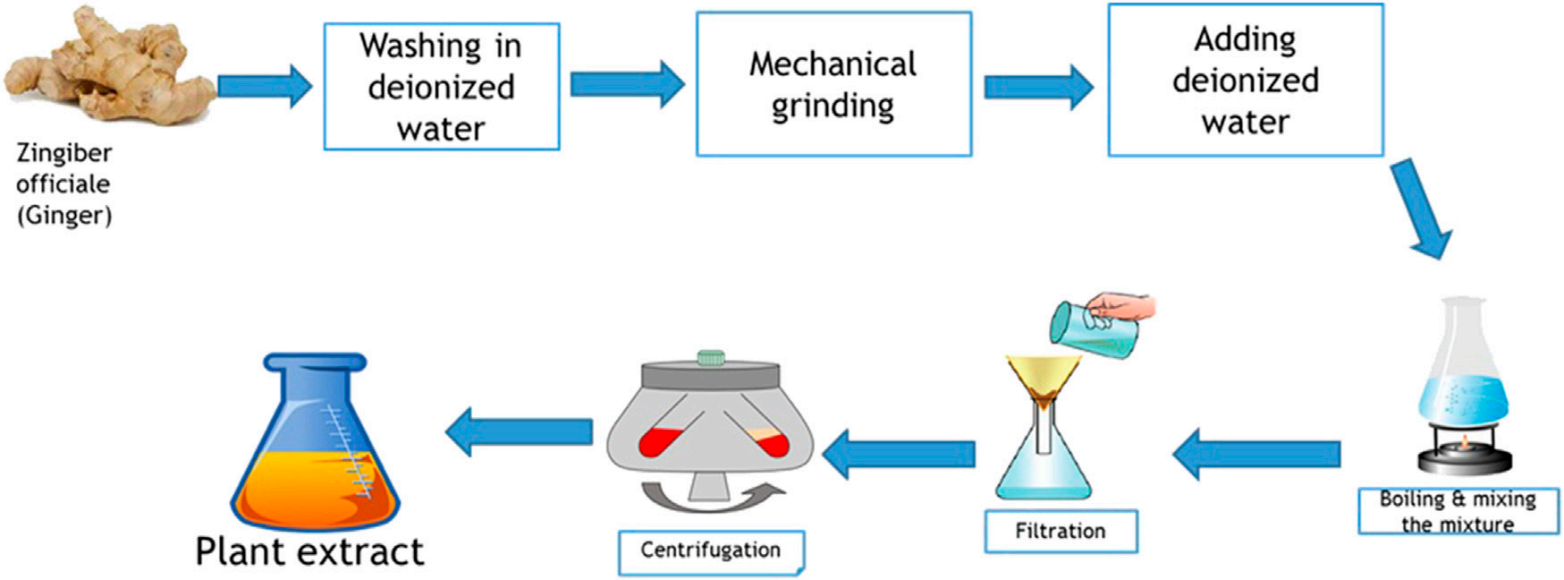
The processes of preparing the extract of ginger.

Fresh *Z. officinale* roots were thoroughly washed with ultra-pure deionized water. After shredding the ginger roots, they were ground using a blender. Subsequently, 20 g of the ground ginger was mixed with 250 mL of deionized water and boiled for 20 min. To obtain the ginger extract, the solution was then filtered using Whatman No. 1 filter paper. The extract was then centrifuged at 6,000 rpm for 10 min. The resulting extract was used as both a reducing and stabilizing agent in the NP synthesis (Figure 1).

##### 3.1.1.2 Biosynthesis of gold nanoparticles using Z. officinale extract

The synthesis of gold nanoparticles involves mixing an aliquot of hydrogen tetrachloroaurate (III) trihydrate (HAuCl_4_.3H_2_O) and an extract of *Z. officinale* in water. A solution of 1 mM of the gold salt was prepared in deionized water. In the next stage, 1 mL of this 1 mM HAuCl4 solution was diluted with deionized water to a final volume of 10 mL and brought to a boil. Then, 1 mL of the ginger extract was added to the boiling solution, and the mixture was kept boiling and stirring at 600 rpm until the solution turned purple. The procedure followed the general approach described by Yadi et al. with modifications including the use of a 1 mM HAuCl4 solution, specific stirring conditions and deionized water as the solvent instead of ethonal (Yadi et al., 2022).

##### 3.1.1.3 Synthesis of gold nanoparticles using pure curcumin

An amount of 3.68 mg of Curcumin with a purity of 95% from *Turmeric rhizome* was dissolved in 2 mL of 10 mM aqueous NaOH solution, and the volume was adjusted to 10 mL with deionized water. To synthesize gold nanoparticles, 1 mL of 1 mM HAuCl_4_ was added to 8 mL of water. To this, 1 mL of the freshly prepared Curcumin solution was added drop wise while stirring in 600 rpm for 20 min. The solution changed colour from yellow to pink (Sreelakshmi et al., 2013).

### 3.2 Chemically synthesis of gold nanoparticles using sodium citrate

AuNps were chemically synthesized by the reduction of HAuCl4 with sodium citrate. About 1 mL of 1 mM HAuCl4 was mixed with 10 mL of deionized water and 1 mL sodium citrate 38.8 mM with vigorous stirring (600 rpm) for 10 min. The mixture was boiled under stirring and then the hot plate was turned off. A color change was observed from colorless to wine red within 10 min and stirring was continued for 20 min (De Souza et al., 2019).

### 3.3 Purification and pH Adjustment of synthesized gold nanoparticles

To purify the synthesized gold nanoparticles from the mixture containing ginger extract, the solution was centrifuged using an ultracentrifuge in four steps. In the first step, centrifugation was carried out at 10,000 rpm for 20 min at 4°C. The supernatant was separated from the settled suspension. Deionized water was added to the pellet, and centrifugation was repeated at 20,000, 30,000, and 40,000 rpm for 20 min each at 4°C. During the purification process, the pH of the solutions was measured using a calibrated pH meter. The pH levels of the ginger extract, curcumin suspension in water, and 1 mM HAuCl₄ solution were recorded. Additionally, the final pH values of both biologically and chemically synthesized AuNPs were also evaluated.

### 3.4 Characterization

The characterization of the synthesized gold nanoparticles (AuNPs) was performed using several analytical techniques. UV-Vis spectroscopy was conducted with a Thermo Scientific Genesys 30 spectrophotometer to identify the characteristic surface plasmon resonance peak, confirming the formation of AuNPs (Burda et al., 2005). Fluorescence measurements were taken using a Horiba FluoroMax4 fluorometer to assess the photoluminescent properties of the nanoparticles (Tiwari et al., 2011). The ginger extract components were analyzed using an Agilent Technologies 5973/6890 GC-MS system, equipped with an HP-5 30 × 0.25 capillary column, to identify the phenolic compounds involved in the reduction process, with the analysis performed at the national library (Yadi et al., 2022) Dynamic Light Scattering (DLS) and zeta potential measurements were carried out using a Malvern instrument to determine the size distribution and stability of the AuNPs (Malvern Instruments). Fourier-Transform Infrared Spectroscopy (FT-IR) was performed with a Nicolet 4700 FT-IR, utilizing 32 scans and a resolution of 4 cm⁻^1^, to identify the functional groups on the AuNPs’ surface (Colthup, 2012). Energy Dispersive X-ray Spectroscopy (EDX) analysis was conducted using a Bruker system to confirm the elemental composition and purity of the AuNPs (Goldstein et al., 2017). Transmission Electron Microscopy (TEM) images were obtained with a Tecnai F20 transmission electron microscope, operating at 200 kV, to visualize the morphology and size of the nanoparticles (Sharma, 2005).

### 3.5 Antimicrobial activity assessment

To assess the antimicrobial activity, AuNPs were passed through a syringe filter with a pore size of 0.45 µm to remove any bacteria/fungi that may have come through the preparation. This process was carried out after measuring the sizes of the gold nanoparticles using dynamic light scattering (DLS).

Bacteria indicator strains of the Gram-positive *Staphylococcus aureus* ATCC 29213 and Gram-negative *Pseudomonas aeruginosa* ATCC 27833 and *Escherichia coli* ATCC 25922 were used to determine antimicrobial activity of gold nanoparticles synthesized with ginger extract, curcumin, and those produced chemically. The minimum inhibition concentration (MIC) values were determined through the microdilution method providing insights into the effectiveness of the nanoparticles in inhibiting bacterial growth (Gouyau et al., 2021).

Additionally, biomolecules from ginger and curcumin were tested to evaluate their impact on antimicrobial activity. This approach helped to understand the combined effect of the biocompounds and gold nanoparticles in suppressing the growth of pathogenic microorganisms.

## 4 Results and discussion

### 4.1 Synthesis of gold nanoparticles using natural extracts and chemical agents

The synthesis of gold nanoparticles was successfully achieved using three distinct methods: ginger extract, curcumin, and sodium citrate. The *Zingiber officinale* extract facilitated a notable color transition from pale yellow to purple, which was confirmed by UV-Vis spectroscopy with a strong absorbance peak at approximately 530 nm, indicating the effective formation of gold nanoparticles primarily within the size range of 10–50 nm. This result suggests that ginger extract not only serves as a reducing agent but also stabilizes the nanoparticles, potentially due to the presence of bioactive compounds such as phenolics and flavonoids. In contrast, the use of curcumin resulted in a change from yellow to pink, with an absorbance peak around 540 nm and a particle size distribution of 20–60 nm. This method underscores curcumin’s dual role as both a reducing and stabilizing agent, further highlighting its potential for eco-friendly nanoparticle synthesis. The chemical synthesis with sodium citrate exhibited a color change from colorless to wine red, confirming nanoparticle formation with a peak around 520 nm and a size distribution of 15–45 nm. These findings indicate that both natural extracts and chemical agents can effectively synthesize gold nanoparticles with varying characteristics, suggesting potential applications in biomedical fields, drug delivery, and catalytic processes.

### 4.2 Physical chemical characterization

#### 4.2.1 Nanomaterial size distribution and surface potentials

**FIGURE 2:**
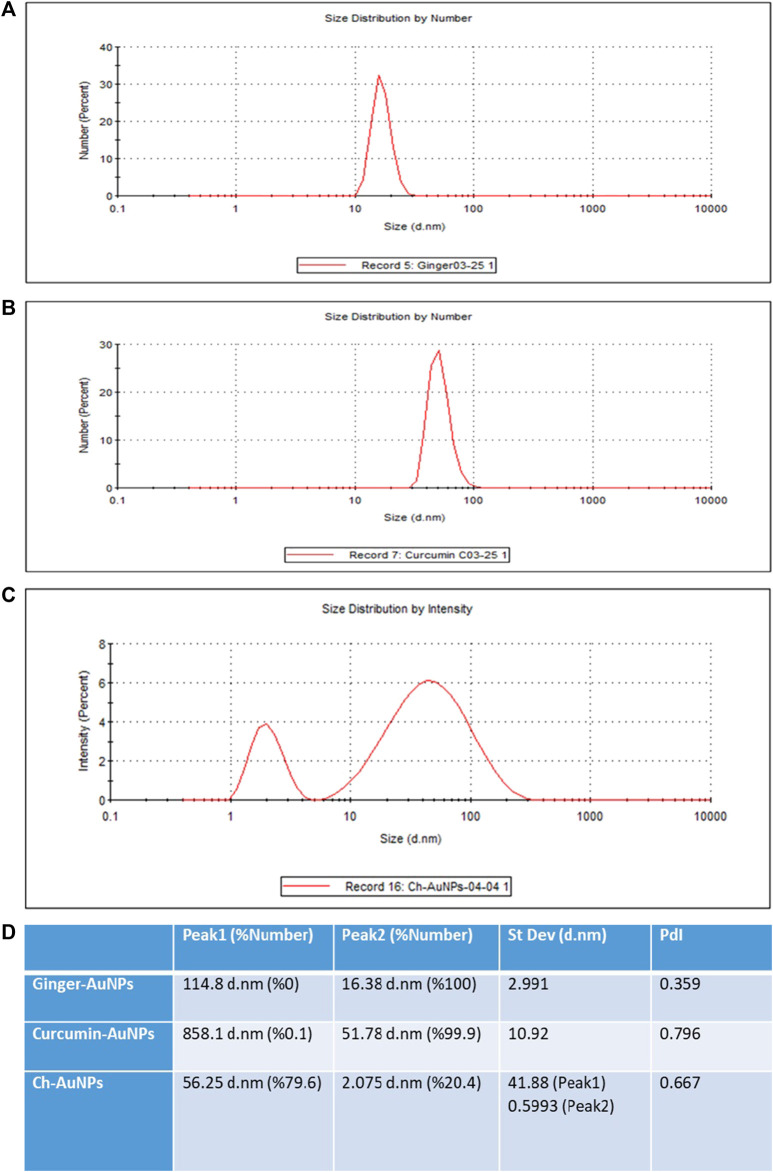
Dynamic light scattering (DLS) analysis AuNPs synthesized with the extract of ginger **(A)**, synthesized AuNPs with curcumin **(B)**, chemically synthesized AuNPs **(C)**, Values of the peaks and their standard deviations and PdIs **(D)**.

**FIGURE 3:**
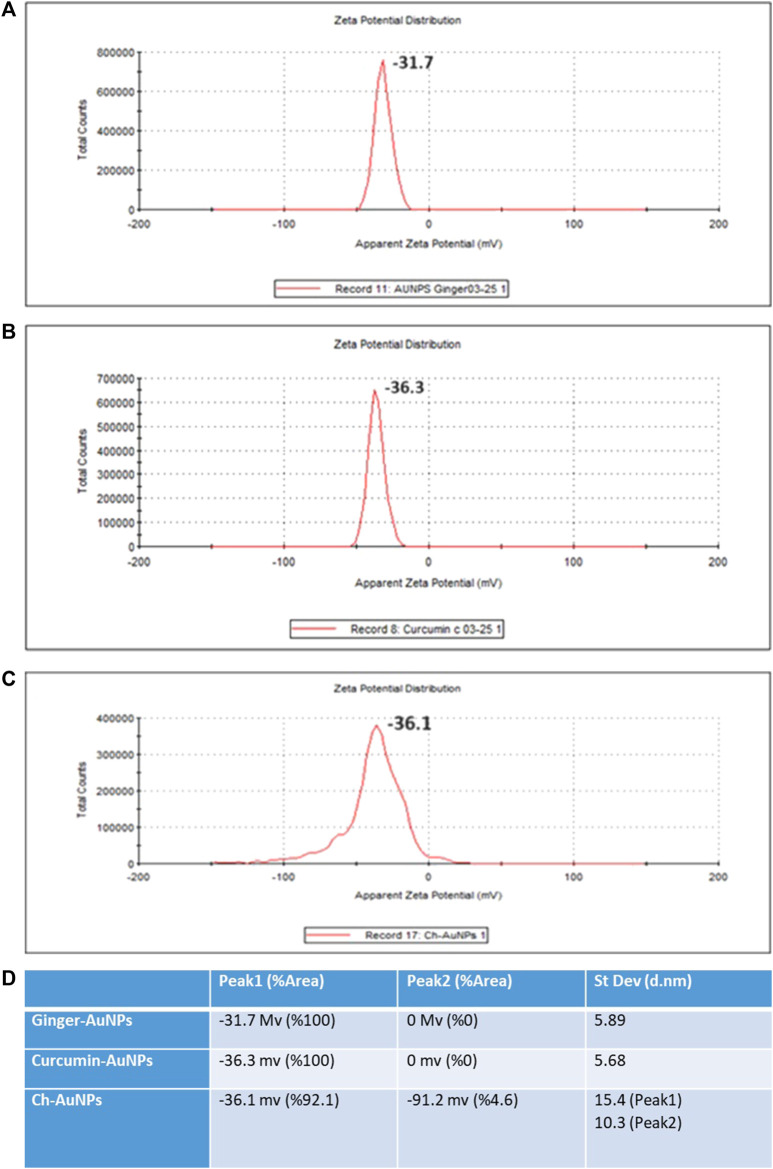
Zeta potential **(A)** Ginger-AuNPs. **(B)** Curcumin-AuNPs. **(C)** Chemical-AuNPs. **(D)** Summary of the results.

The hydrodynamic diameter of biosynthesized gold nanoparticles with ginger extract, curcumin gold nanoparticles, and chemically synthesized gold nanoparticles using sodium citrate has been evaluated by dynamic light scattering (DLS) analysis (Figure 2). Biosynthesized AuNPs with ginger extract are characterized by a sharp peak with an average particle size of 16.83 nm (Figure 2A). The curcumin gold nanoparticles show a sharp peak with an average size of 51.78 nm (Figure 2B), while the chemically synthesized gold nanoparticles exhibit two broad peaks: one with a diameter of 56.25 nm and 79.6% intensity, and another with a diameter of 2.07 nm and 20.4% intensity (Figure 2C). The size distribution of biosynthesized gold nanoparticles using ginger extract and curcumin is clearly superior to that of chemically produced AuNPs, which is also reflected in the polydispersity index (PDI) of each sample (Figure 2D). This superior size distribution leads to better stability and functionality, critical for biomedical applications (Huang and El-Sayed, 2010). Moreover, Zeta potential measurements have been conducted to study the stability of biosynthesized gold nanoparticles with ginger extract, curcumin gold nanoparticles, and chemically synthesized AuNPs in solution. In all cases, a negative zeta potential value was detected (Figure 3). Specifically, green-synthesized AuNPs with ginger extract exhibited a surface charge of −31.7 mV (Figure 3A). The surface charge of curcumin AuNPs was −36.3 mV (Figure 3B), while chemically synthesized AuNPs showed a surface charge of −40.4 mV (Figure 3C). A zeta potential of at least ± 20 mV gives good thermodynamic stability, making these particles useful for a number of biomedical applications (Honary and Zahir, 2013). The higher stability observed in the biosynthesized nanoparticles could enhance their performance in therapeutic and diagnostic applications, reducing aggregation and improving bioavailability (Vadlapudi and Kaladhar, 2014) These findings underscore the potential of biosynthesized gold nanoparticles, particularly those synthesized with natural extracts like ginger and curcumin, in enhancing the efficacy of nanoparticle-based biomedical applications. The stability and size distribution advantages they offer are critical for their integration into multifunctional platforms for targeted drug delivery and advanced diagnostics (Sperling and Parak, 2010).

#### 4.2.2 Electron microscopy

**FIGURE 4:**
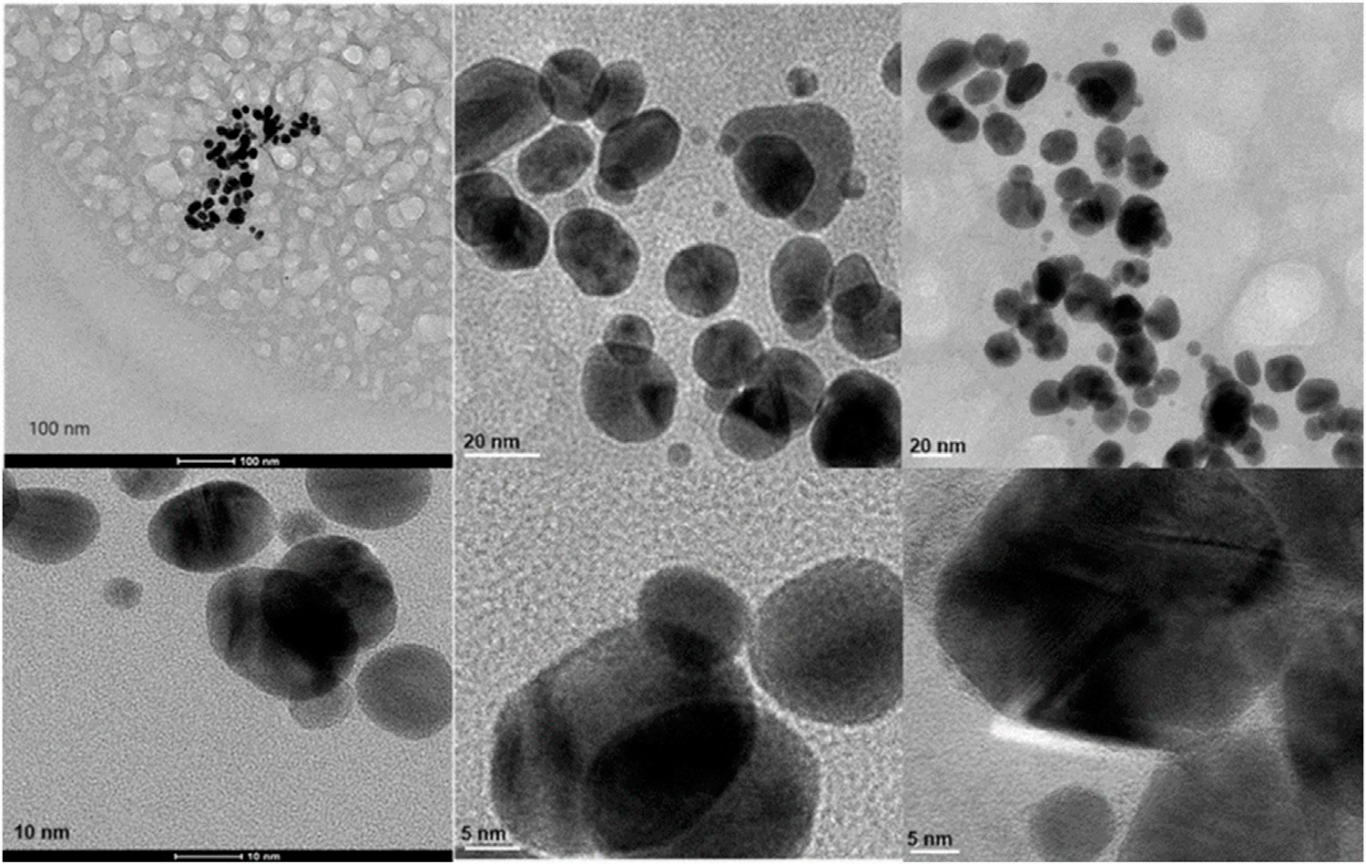
Transmission electron microscopy figures under different magnifications and field views for biosynthesized AuNPs with ginger extract.

**FIGURE 5:**
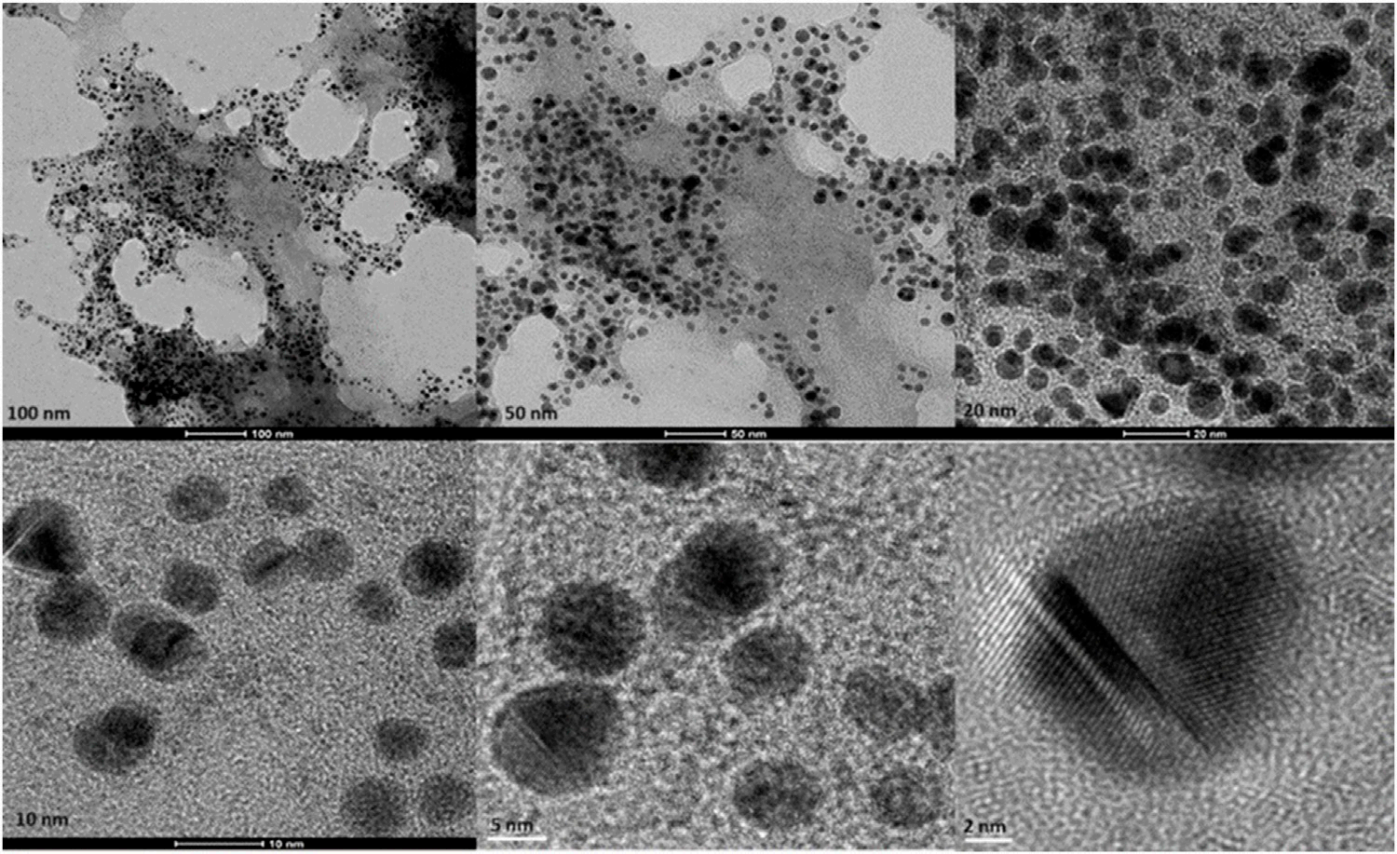
Transmission electron microscopy figures under different magnifications and field views for biosynthesized AuNPs with curcumin.

**FIGURE 6:**
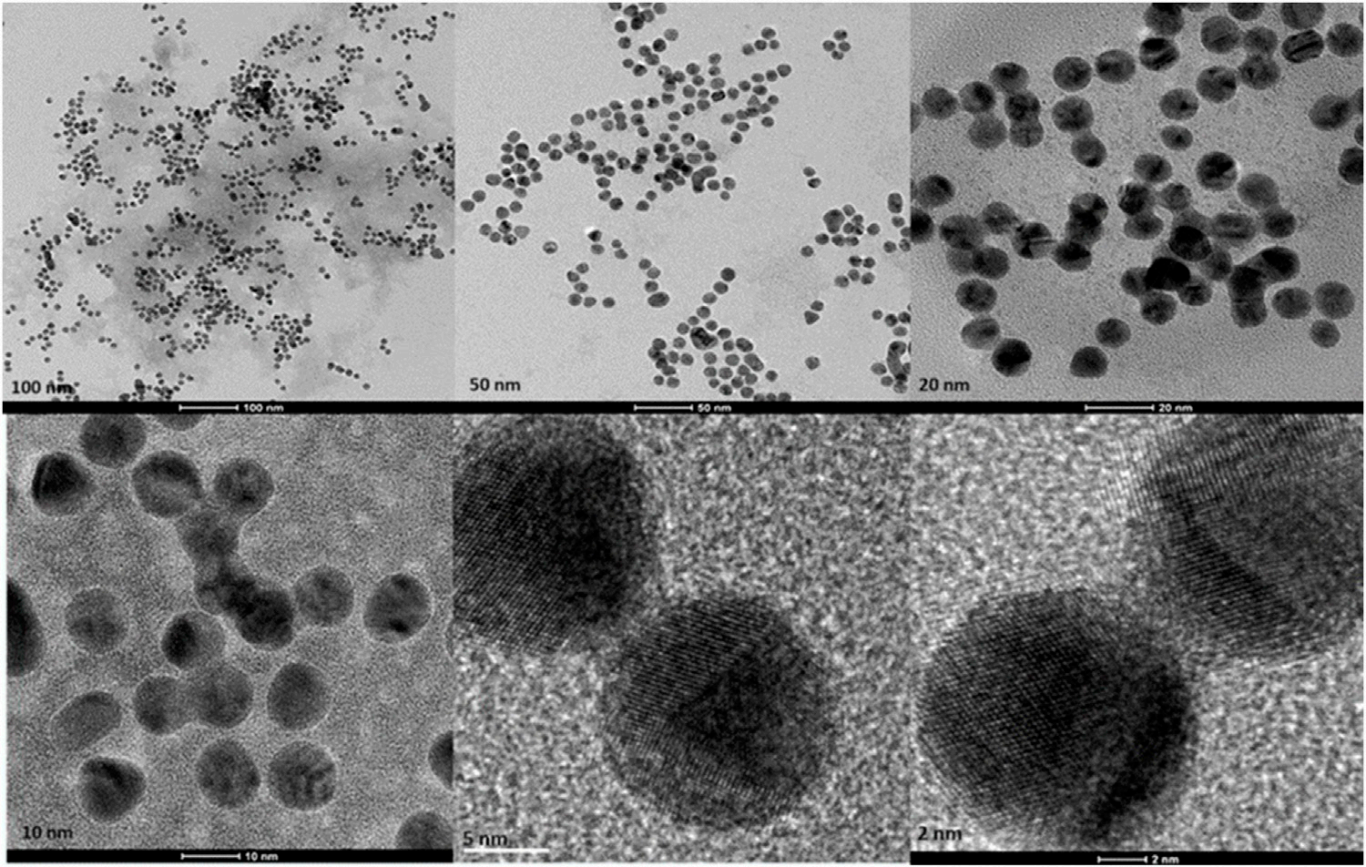
Transmission electron microscopy figures under different magnifications and field views for chemically produced AuNPs.

Transmission Electron Microscopy (TEM) images of biologically and chemically synthesized AuNPs, ranging in size from 2 to 100 nm, revealed various features of the different synthesis methods and the structures of the formed nanoparticles. The TEM images in Figure 4 show spherical and oval-shaped gold nanoparticles produced with ginger extract, with an average size of 20 nm. At lower magnification, biological materials are visible as background vesicles, while at higher magnifications, the lattice structure of the gold nanoparticles is evident. This indicates a high degree of crystallinity in the biosynthesized nanoparticles (Narayanan and Sakthivel, 2010). Figure 5 reveals spherical biosynthesized curcumin gold nanoparticles with an average size of 10 nm. Since pure curcumin was used as the biological material, there are no traces of cells or large biological components, yet the particles are still surrounded by the biological material, likely contributing to their stability and dispersity in solution (Dykman and Khlebtsov, 2011). Figure 6 presents spherical chemically produced gold nanoparticles with an average size of 10 nm. These nanoparticles exhibit a more uniform shape and size distribution compared to biosynthesized ones, which can be attributed to the controlled conditions of chemical synthesis (Daniel and Astruc, 2004) However, in agreement with the dispersion of sizes from the DLS, many of the particles seem fused together. The differences in morphology and size between biologically and chemically synthesized nanoparticles highlight the influence of the synthesis method on nanoparticle characteristics, which in turn affects their potential applications in biomedicine (Bhattacharya and Mukherjee, 2008).

#### 4.2.3 Elemental compositions of gold nanoparticles

**FIGURE 7:**
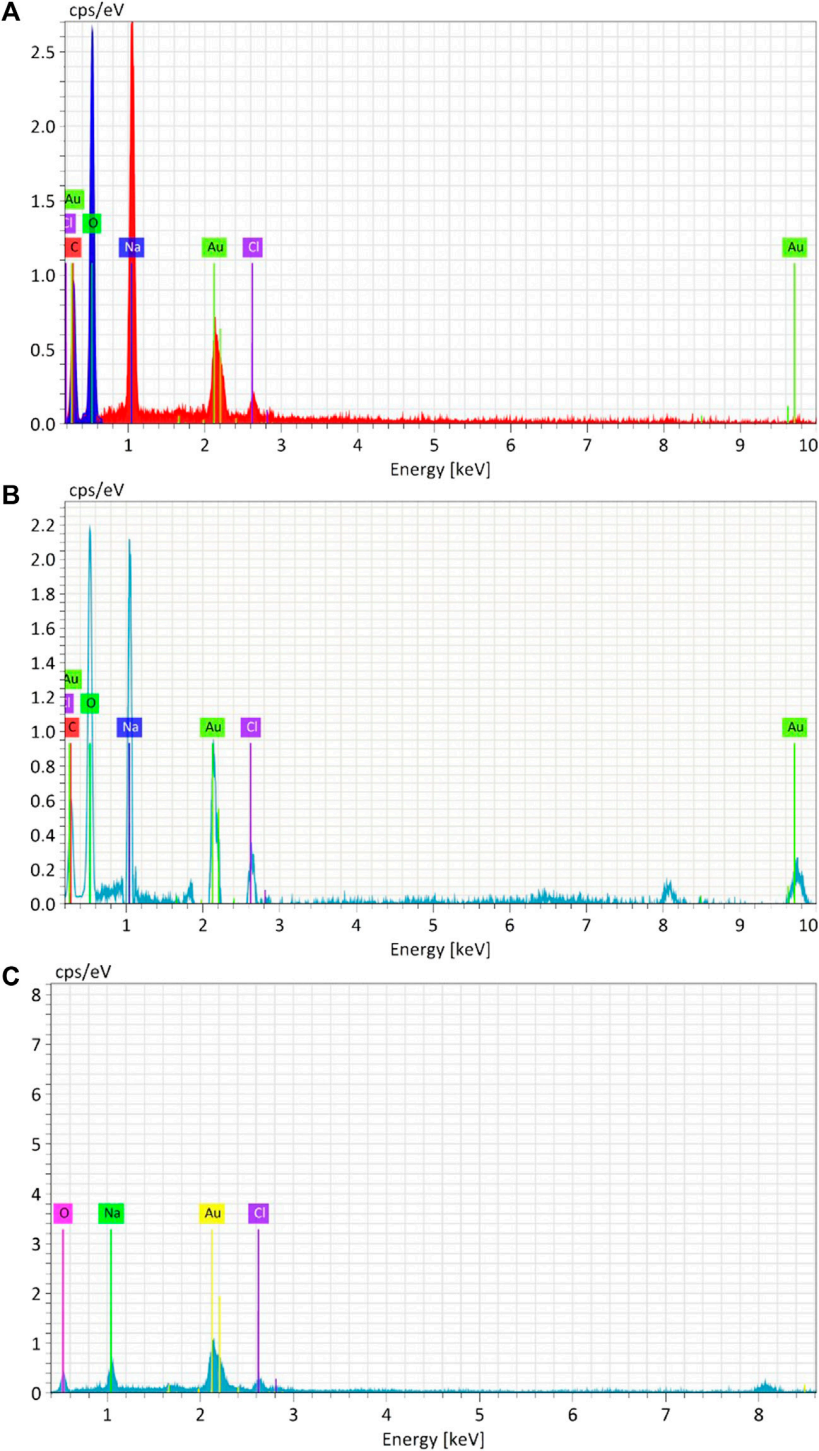
Energy-dispersive X-ray analysis (EDAX) of Synthesized AuNPs with Ginger extract **(A)**, Curcumin **(B)** and chemically produced nanoparticles with Sodium citrate **(C)**.

The elemental composition of biosynthesized and chemically produced AuNPs was assessed through energy-dispersive X-ray spectroscopy (EDAX) (Figure 7). Analysis of the biosynthesized and chemical AuNPs samples revealed the presence of Au, with peaks at 2.2 keV. Both biological AuNPs exhibited similar compositions, including the presence of carbon and oxygen. However, in the chemical AuNPs, there were no strong peaks of C and O. This indicates that the strong carbon and oxygen peaks in the biological AuNPs are associated with the organic molecules likely comprising their cap. Additionally, the TEM observations suggested the presence of organic material surrounding the nanoparticles. This suggests that the biological synthesis method results in nanoparticles capped with organic materials, which can enhance their stability and biocompatibility (Saha et al., 2012).

The presence of carbon and oxygen in biosynthesized nanoparticles can be attributed to the phytochemicals from the ginger extract and curcumin, which act as both reducing and capping agents. These organic compounds not only stabilize the nanoparticles but also provide functional groups that can be further utilized for bioconjugation in biomedical applications (Ahmed et al., 2016).

### 4.3 Absorption spectrum of AuNPs

**FIGURE 8:**
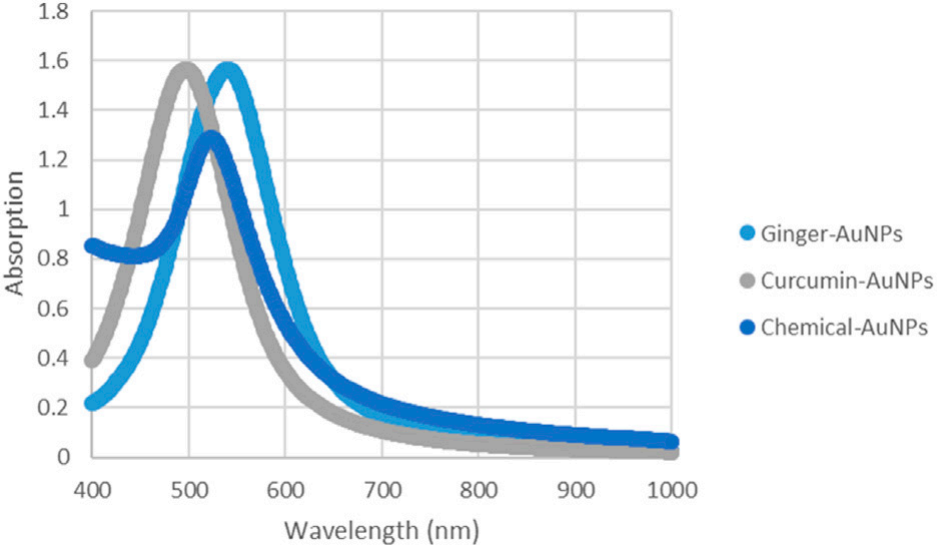
UV-vis Absorption spectra of AuNPs synthesized with ginger extract, Curcumin AuNPs and chemically produced AuNPs.

UV-Vis absorption spectroscopy is a crucial technique for evaluating the synthesis of metal nanoparticles in aqueous solutions (Figure 8). The stability is indicated by the maximum absorbance value remaining constant when samples are analyzed over different time periods (Chanana and Liz-Marzan, 2012). According to the charts, the UV-Vis spectra measurements of biosynthesized gold nanoparticles with ginger extract and curcumin, as well as chemically produced AuNPs, show a peak around 550 nm. This peak is characteristic of the surface plasmon resonance (SPR) of gold nanoparticles, which is typically observed in the range of 500–550 nm, depending on the size and shape of the nanoparticles (Haiss et al., 2007). The presence of a consistent peak over time in the UV-Vis spectra indicates that the nanoparticles are stable in solution without significant aggregation or shape change, which is critical for their potential biomedical applications (Dreaden et al., 2012).

### 4.4 Fluorescence spectroscopy of the gold nanoparticles

**FIGURE 9:**
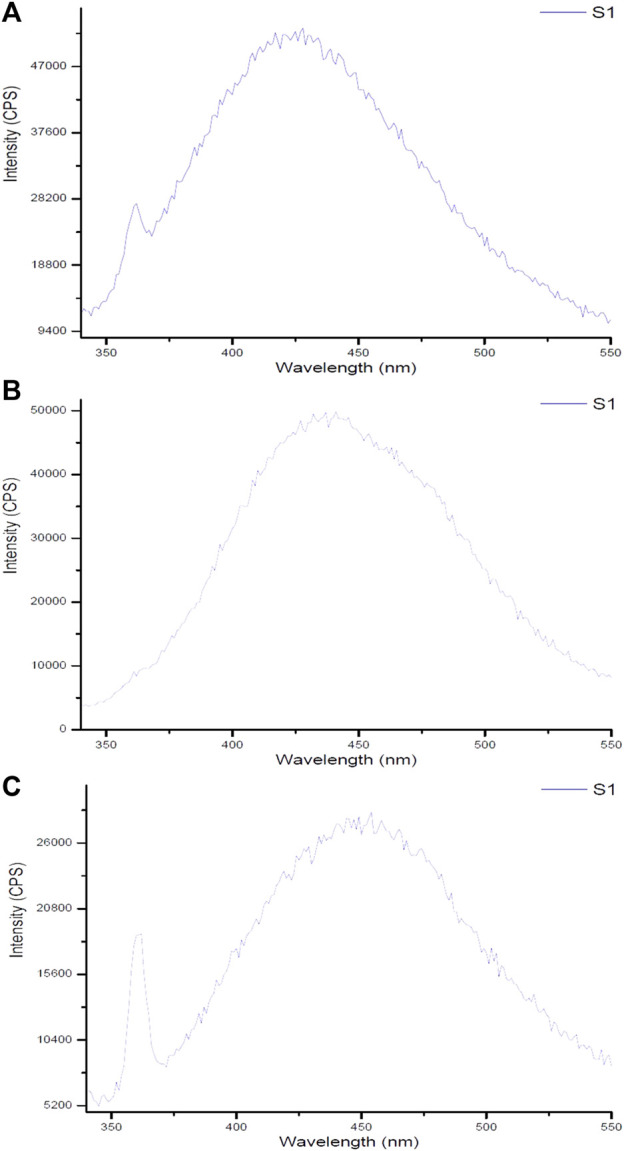
Fluorescent spectra of gold nanoparticles synthesized using **(A)** ginger extract, **(B)** curcumin, and **(C)** chemical method.

The spectrum of Ginger-AuNPs shows a broad peak, indicating successful synthesis and characteristic optical properties of AuNPs (Figure 9). This broad peak is typically associated with the localized surface plasmon resonance (LSPR) of gold nanoparticles, which is consistent with observations in other studies of gold nanoparticle synthesis using plant extracts such as ginger (Yadi et al., 2022). The higher intensity observed in the fluorometer results for Curcumin-conjugated AuNPs suggests a strong interaction and successful reduction of gold ions by curcumin. This is supported by our data here indicating that curcumin acts effectively as both a reducing and capping agent, leading to stable gold nanoparticles with enhanced optical properties. The successful formation of these nanoparticles are evident in their intense fluorescence, which aligns with previous studies on curcumin gold nanoparticles (Sindhu et al., 2014). In comparison, the results for chemically synthesized AuNPs show lower intensity than the green-synthesized AuNPs. However, they still exhibit typical optical properties of gold nanoparticles, such as the LSPR peak, which confirms the formation of gold nanoparticles albeit with less pronounced optical characteristics compared to their biologically synthesized counterparts. This difference in intensity and optical properties between green-synthesized and chemically synthesized AuNPs can be attributed to the presence of organic molecules in the green synthesis process, which can enhance the stability and functional properties of the nanoparticles (Yadi et al., 2022; Link and El-Sayed, 2003).

Monitoring fluorescence offers valuable insights into the stability of nanoparticles under various environmental conditions. As demonstrated by Pallem et al. (2009), the fluorescence properties of gold nanoparticles are significantly influenced by their size, shape, and surface functionalization, which are critical for determining their stability in solution. Moreover, excitation-emission fluorescence data can act as a sensitive indicator of nanoparticle morphology, revealing structural changes over time or in response to external factors. The results from fluorescence measurements of synthesized gold nanoparticles using biological compounds such as ginger and curcumin, compared to chemically produced counterparts, indicate that the biologically synthesized nanoparticles exhibit superior size and morphology, enhancing their stability. Although some noise in the fluorescence data was observed—attributed to the water solvent used during synthesis—this also highlights the environmentally friendly conditions under which these nanoparticles were produced. The use of water emphasizes the intrinsic properties of the synthesized gold nanoparticles and their potential efficacy in combating bacteria. These findings underscore the advantages of biological synthesis methods for developing stable materials suitable for various applications.

### 4.5 Organic material characterization

**FIGURE 10:**
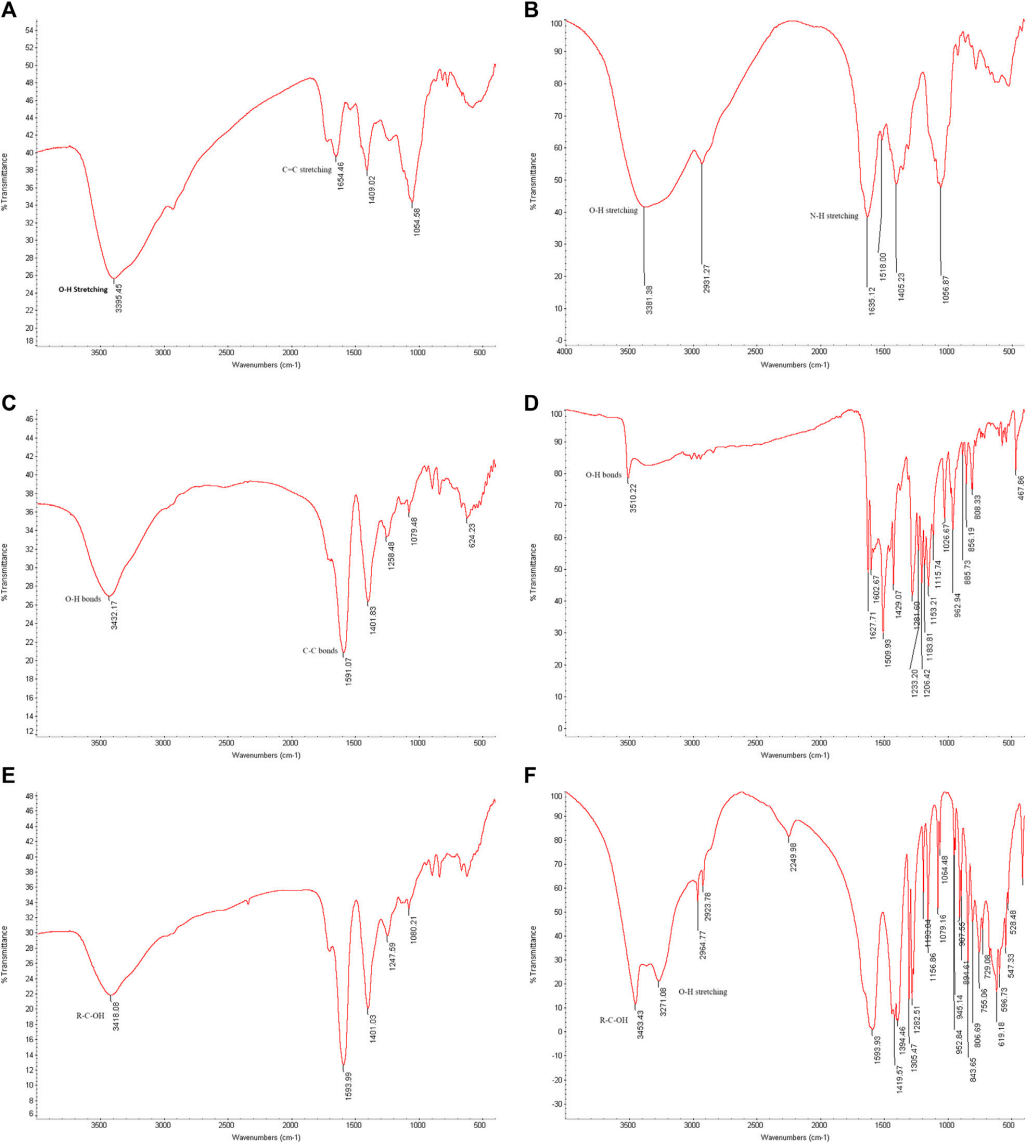
FTIR spectrum. **(A)** Biosynthesized AuNPs with ginger extract. **(B)** Ginger root extract. **(C)** Biosynthesized AuNPs with Curcumin, **(D)** Pure Curcumin. **(E)** Chemically produced AuNPs with sodium citrate. **(F)** Sodium citrate.

To analyze the gold nanoparticles using FTIR spectra, the samples were freeze-dried. The spectra results provided information about the cap compositions of the gold nanoparticles. Figure 10 (A) shows the FTIR spectra results of biosynthesized gold nanoparticles using ginger root extract, with a peak at 3,395 cm⁻^1^ attributed to the O-H stretching of phenolic compounds. The band at 1,654 cm⁻^1^ corresponds to the C=C stretching of alkenes. Figure (B) illustrates the functional group of the ginger extract, where the signal at 3,381 cm⁻^1^ is associated with O-H bonds of phenolic compounds, and the signal at 1,635 cm⁻^1^ is related to N-H stretching of amides. Figure (C) presents the FTIR spectra of the biosynthesized AuNPs with Curcumin, showing a signal at 3,432 cm⁻^1^ due to the O-H bonds of phenolic compounds. Additionally, the sharp peak at 1,591 cm⁻^1^ corresponds to the C-C bonds of aromatic compounds. In Figure(D), pure Curcumin displays a signal at 3,510 cm⁻^1^ due to its O-H bonds, with two other signals at 1,627 and 1,602 cm⁻^1^ corresponding to C=O bonds. The sharp peak at 1,509 cm⁻^1^ indicates the C=C bonds of Curcumin. Figure (E) reveals the components associated with chemically produced AuNPs, with signals at 3,418 cm⁻^1^ due to R-C-OH groups. In Figure (F), the signals at 3,453 and 3,271 cm⁻^1^ are attributed to the OH group of sodium citrate. It is interesting that the biomolecules when associated with the AuNPs provide a more simplified and peak broadened spectrum, which might be the result of the influence of surface plasmons.

**FIGURE 11:**
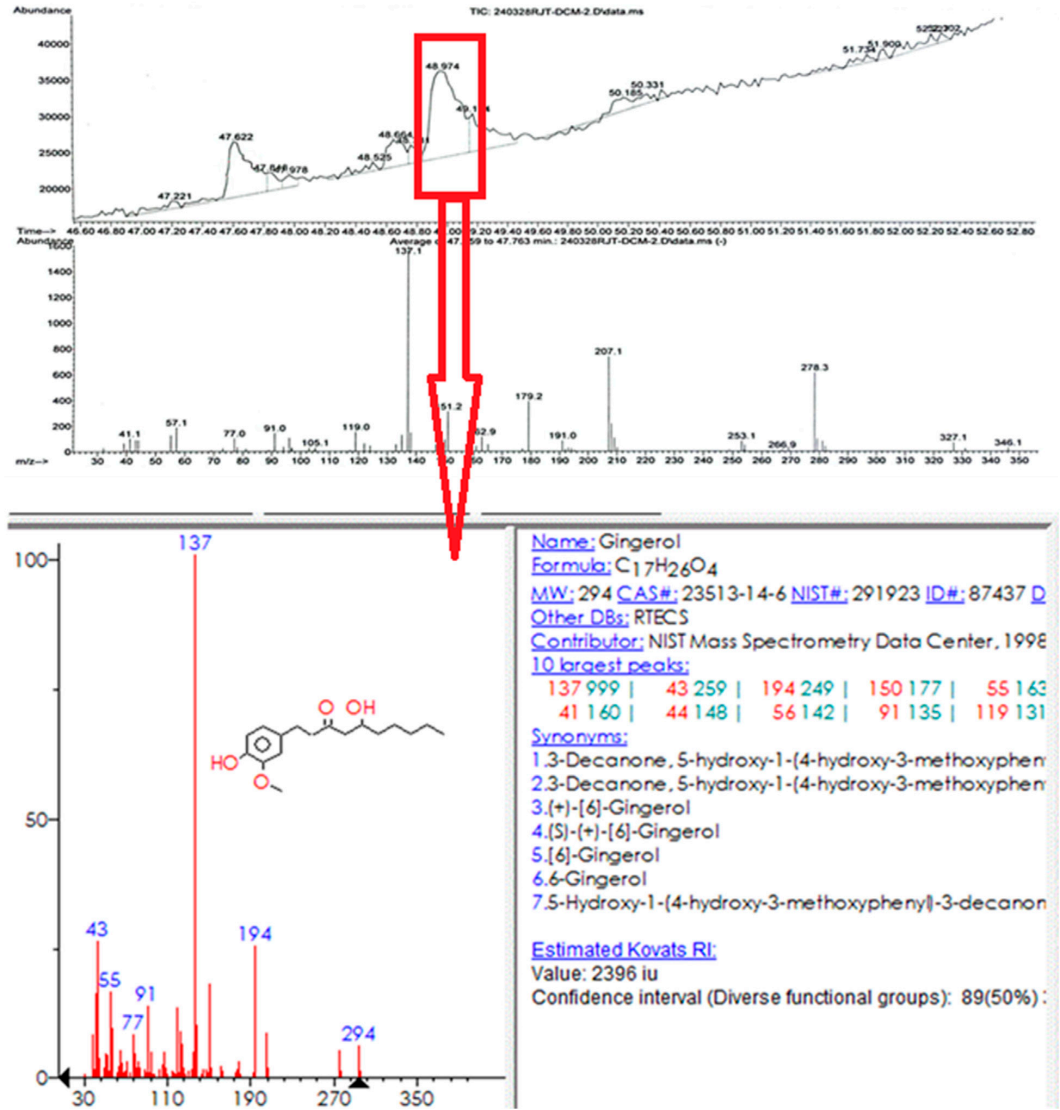
GC-MS analysis of the extract of ginger root.

Ginger extract was analyzed using gas chromatography-mass spectrometry (GC-MS). Figure 11 shows a graph with a strong signal at 48.974. This signal was compared with mass spectra patterns from the standard library data of the National Institute of Standards and Technology (NIST). The library search analysis indicates that the signal can be associated with Gingerol. Since Gingerol is a phenolic phytochemical compound and its strongest signals are due to its high concentration in the extract and considering the FTIR spectra analysis of the biosynthesized AuNPs, which shows that phenolic groups are associated with the AuNPs, it suggests that Gingerol is highly likely to be present in the caps of the gold nanoparticles.

GC-MS was chosen for the analysis because ginger root contains many volatile substances, making it more suitable than LC-MS, which is typically used for high molecular weight compounds. Since water was used as the solvent and the extract was filtered before GC-MS analysis, the baseline was understandably weaker. However, the strongest signal was attributed to the presence of gingerol. It is important to note that using a different solvent might have resulted in the detection of hydrophobic compounds, but in this case, hydrophilic compounds were predominantly observed.

### 4.6 Effect of NP Purification on pH

The biologically and chemically synthesized gold nanoparticles (AuNPs) were evaluated for pH changes resulting from the synthesis and purification process. Table 1 shows the initial pH levels of the synthesized AuNPs without purification, which could influence the outcomes of antimicrobial activity assessments. The pH assessment revealed that the ginger extract and curcumin suspension had a neutral pH of around 6.5. The pH of 1 mM HAuCl₄ was strongly acidic, which lowered the overall pH of the solution. Although the pH of 3.86 mM sodium citrate was basic at approximately 8.0, its minimal addition did not significantly alter the acidic nature of the HAuCl₄ solution. The purification process, including centrifugation and washing with deionized water, led to an increased pH of the purified AuNPs. The assessment of pH before and after purification revealed significant changes, which are crucial as they can influence the antimicrobial activity of the gold nanoparticles. Understanding these pH variations is essential for accurately interpreting the efficacy of the nanoparticles.

**TABLE 1:** pHs of AuNP mixtures.

	Non-purified AuNPs			Purified AuNPs		
NPs	Ginger-AuNPs	Curcumin-AuNPs	Chemical AuNPs	Ginger-AuNPs	Curcumin-AuNPs	Chemical AuNPs
pHs	5.5	5.3	5.2	6.4	6.3	6.3

### 4.7 Antimicrobial activity

The efficacy of biosynthesized gold nanoparticles (AuNPs) with ginger extract and curcumin, compared to chemically produced AuNPs, was tested against planktonic cells of clinical isolates of *P. aeruginosa, E. coli,* and *S. aureus*. To evaluate the effect of biological materials and chemical reagents on antimicrobial activity, the materials, gold salt, and the mixture of HAuCl_4_ with the biological reagents before the formation of gold nanoparticles were assessed (Table 2). The results demonstrated that curcumin AuNPs exhibited strong antimicrobial activity compared to ginger-AuNPs and chemically produced gold nanoparticles. However, green synthesized AuNPs with ginger showed better activity than chemically produced AuNPs. The concentrations of ginger extract (0.8 mg/mL) and curcumin (0.36 mg/mL) used for synthesizing gold nanoparticles did not exhibit any antimicrobial activity when tested on their own at the same concentrations used in the nanoparticle synthesis.

**TABLE 2:** Minimum Inhibition Concentration (MIC) and Minimum Bactericidal Concentration (MBC) assays of *Pseudomonas aeruginosa*, *Staphylococcus aureus* and *Escherichia coli.*

MIC and MBC (mg/ml^−1^) Bacteria Strains						
	*P. aeruginosa*		*S. aureus*		*E. coli*	
	MIC	MBC	MIC	MBC	MIC	MBC
Ginger-AuNPs	1.4	2.8	1.4	1.4	2.8	2.8
Curcumin-AuNPs	1.4	2.8	1.4	1.4	2.8	2.8
Ch-AuNPs	2.8	2.8	2.8	2.8	2.8	2.8

### 4.8 Comparative analysis

Our AuNPs synthesized with ginger root extract and curcumin exhibited well-defined peaks in the FTIR spectra, indicating successful capping by organic molecules. The purification process effectively removed biological residues and salts, and the nanoparticles were freeze-dried to prevent water-related artifacts. This led to clear and interpretable FTIR spectra, with minimal interference.

Here we compare to other biogenic synthesis of AuNPs compiled in Table 3. Yang et al. (2014) synthesized AuNPs using waste mango peel extract, resulting in particles ranging from 6 to 18 nm. However, their FTIR analysis was less precise, likely due to the lack of a purification process and freeze-drying. As a result, water molecules surrounding the nanoparticles introduced unwanted signals in their spectra. Additionally, the absence of zeta potential measurements limited the evaluation of nanoparticle stability (Yang et al., 2014).

**TABLE 3:** Summary of comparative studies on gold nanoparticles synthesized from various plant extracts.

Study	Plant source	Synthesis time	Nanoparticle size (nm)	Shape distribution	Zeta potential (mV)	Key limitations	Our study
Paul et al. (2015)	*Pogestemon benghalensis*	12 h	10–50	Spherical, triangular, hexagonal	Not provided	Poor shape and size distribution; lack of zeta potential data	10–20 nm with uniform shape; zeta potential −30 to −40 mV
Patra et al. (2015)	*Butea monosperma*	24 h	10–100	Various shapes (spherical, rods, triangular, hexagonal)	−12.8 to −16.4	Limited geographic availability; weak stability indicated by low zeta potential	Widely available ginger and curcumin; zeta potential −30 to −40 mV
Adewale (2020)	*Crassocephalum rubens*	Not specified	Not specified	Not specified	Not provided	Limited geographic availability; lack of zeta potential and DLS measurements	Included zeta potential and DLS measurements
Nadagouda et al. (2014)	Blackberry, blueberry, pomegranate, turmeric	Overnight	>100	Aggregated	Not provided	Long synthesis time; unspecified stirring speed; poor size distribution and stability	Time-efficient and clearly specified parameters; no aggregation
Aljabali et al. (2018)	*Ziziphus zizyphus*	Not specified	Not specified	Triangular, hexagonal	Not provided	Heterogeneous nanoparticles; no antimicrobial activity at higher concentrations	Significant antimicrobial activity at 1.4 mg/mL
Yadi et al. (2022)	*Zingiber officinale (Ginger)*	Not specified	314	Not specified	−7.11	Did not assess ethanol’s effect; no purification process; weak stability	Water extraction and rigorous purification; nanoparticles 10–20 nm

Chandan Tamuly (2013) biosynthesized AuNPs using Gymnocladus assamicus leaf extract, but the synthesis took 4 h under stirring, making it less time-efficient. The UV-Vis spectra revealed broad peaks, likely due to residual biological compounds. TEM images showed aggregation, suggesting poor stability, and the study lacked DLS and zeta potential measurements, leaving size distribution and stability uncertain (Tamuly et al., 2013).

In contrast, our study demonstrated better results in UV-Vis spectra due to proper purification, which removed residual biological compounds. Our TEM images showed no aggregation, and the zeta potential measurements indicated a stable colloidal solution with values greater than −30 mV, confirming better size distribution and stability (Fourt, 1968; Heimenz, 1978). Also Singh et al. (2013) showed that curcumin capped gold nanoparticles have better size distribution and stability for several months that also had a superior antioxidant activity.

In other study, Wu et al. (2013) biosynthesized AuNPs using *Cacumen platycladi* leaf extract. However, the age and type of leaves (seasonality) were not specified, which raises concerns about reproducibility. The nanoparticles were of mixed shapes, including nanospheres and nanoplates, and their sizes were inconsistent, indicating poor control over shape and size distribution (Wu et al., 2013). In our study, using ginger extract and curcumin, we achieved well-controlled and uniform nanoparticle sizes, with excellent shape distribution, making the nanoparticles suitable for various applications. Our method is highly repeatable due to its reliance on globally available plant extracts.

In 2015, Paul et al. (2015) synthesized AuNPs from *Pogestemon benghalensis* leaf extract, resulting in nanoparticles of various shapes (spherical, triangular, hexagonal) and sizes ranging from 10–50 nm. The lengthy 12-hour synthesis time and variable shape distribution indicated limitations in their method, compounded by a lack of zeta potential data, which is crucial for assessing stability. In contrast, our method produced nanoparticles with uniform shape distribution, measuring 10–20 nm (Paul et al., 2015).

Moreover, in the same year, Sujata Patra et al. focused on AuNPs synthesized from *Butea monosperma* leaf extract. The limited geographic availability of this plant limits the reproducibility of their findings, especially outside South and Southeast Asia. Their method required 24 h of stirring, yielding nanoparticles with varying shapes and sizes (10–100 nm) and low zeta potential values (−12.8 to −16.4 mV), suggesting weak stability. Our approach, using widely available ginger and curcumin, resulted in nanoparticles with high surface charge and zeta potential values of −30 to −40 mV, indicating superior stability and no signs of aggregation (Patra et al., 2015).

Adewale et al. (2020) synthesized AuNPs using Crassocephalum rubens leaf extract, which presents challenges for replication in areas like Canada due to its specific geographic distribution. Their study did not include zeta potential or DLS measurements, hindering the assessment of stability and size distribution. Our work, however, incorporated both measurements, confirming high stability and controlled size distribution of our nanoparticles. Furthermore, we highlighted the role of biological compounds in stabilizing AuNPs, which enhanced antimicrobial activity compared to chemically synthesized counterparts (Adewale et al., 2020).

Nadagouda et al. (2014) utilized extracts from various fruits to synthesize AuNPs, with the synthesis process extending overnight and lacking specified stirring conditions that shows the unrepeatability of their methods. Their TEM images revealed particles exceeding 100 nm in size, with evident aggregation, indicating poor size distribution and stability (Nadagouda et al., 2014). In contrast, our method is efficient and specifies clear reaction parameters, resulting in nanoparticles with superior size distribution and stability, confirmed by zeta potential analysis.

Aljabali et al. (2018) synthesized AuNPs from Ziziphus zizyphus leaf extract, which resulted in heterogeneous nanoparticles that did not exhibit antimicrobial activity even at higher concentrations (5 mg/mL). Conversely, our study showed that AuNPs synthesized with ginger extract and curcumin demonstrated significant antimicrobial activity at much lower concentrations of 1.4 mg/mL, emphasizing the effectiveness of our method (Aljabali et al., 2018).

Yadi et al. (2022) prepared AuNPs from ginger root extract using ethanol, but they did not assess the impact of ethanol on antimicrobial efficacy. Their reported particle size was 314 nm, with a zeta potential of −7.11 mV, indicating poor stability (Yadi et al., 2022). Our study, which utilized water for the extraction process and included rigorous purification steps, produced AuNPs ranging from 10–20 nm with a zeta potential between −30 to −40 mV. This approach allowed us to clearly demonstrate the antimicrobial properties attributed to the AuNPs alone, without interference from residual compounds.

## 5 Conclusion

This study successfully demonstrated the synthesis and characterization of gold nanoparticles using both eco-friendly and chemical methods. The use of ginger extract and curcumin as reducing agents resulted in nanoparticles with enhanced stability, uniform size distribution, and significant antimicrobial properties compared to chemically synthesized AuNPs. Notably, the presence of biomolecules in the biosynthesized nanoparticles suggests potential advantages in biocompatibility and functionality, which are crucial for biomedical applications.

The findings underscore the potential of green-synthesized AuNPs, particularly those associated with bioactive compounds like curcumin, in developing effective antimicrobial agents. Future studies could explore the mechanistic aspects of nanoparticle-biomolecule interactions and further evaluate the *in vivo* efficacy and safety of these green-synthesized nanoparticles in medical applications.

## Data Availability

The original contributions presented in the study are included in the article/supplementary material, further inquiries can be directed to the corresponding author.
